# A Gustatory Receptor Used for Rapid Detection of *Tyrophagus putrescentiae* in Fungi Hosts

**DOI:** 10.1038/s41598-018-29729-4

**Published:** 2018-07-30

**Authors:** Shao-Xuan Qu, Xiao-Fei Wang, Hui-Ping Li, Xin Luo, Lin Ma

**Affiliations:** 1 Institute of Vegetable Crops, Jiangsu Key Laboratory for Horticultural Crop Genetic Improvement, Jiangsu Academy of Agricultural Sciences, Nanjing, 210014 China; 2 Cold Spring Harbor Laboratory, 1 Bungtown Rd, Cold Spring Harbor, New York 11724 USA

## Abstract

The storage mite, *Tyrophagus putrescentiae*, found worldwide in many habitats, is an important pest of edible fungi in China. Storage mites are tiny and difficult to observe, especially when they occur in fungi composts. In this study, one gustatory receptor protein (TputGR1) was identified from the transcriptome of *T. putrescentiae*. Phylogenetic analysis of GRs families from 10 arthropod species revealed that TputGR1 had high homology with the SccaGR1 of Sarcoptes scabiei and TurtGR1-2 of *Tetranychus urticae*, but low homology with other insect species, *Drosophila melanogaster*, *Anopheles gambiae*, *Bombyx mori*, *Aedes aegypti*, *Culex quinquefasciatus*, and *Pediculus humanus*. We developed a detection system for the mite on fungi hosts using the GR protein and the loop-mediated isothermal amplification (LAMP). This procedure was rapid (60 min from sampling to result) and had high sensitivity (0.5 ng/mL). LAMP provided rapid and reliable detection of *T. putrescentiae*. It has good specificity for single samples and for large-scale surveys.

## Introduction

The storage mite, *Tyrophagus putrescentiae*, is one of the most common synanthropic mites. It has a wide range of habitats, including stored products, crop seeds, mushrooms, foods of domestic animals and pets, and house dust^1^. It also causes human allergic dermatitis^2^, pulmonary acariasis and intestinal acariasis^3^. Fungi have large amounts of nitrogen and phosphorus and produce vitamins and sterols, which are important for mite development^4^. Fungi also produce exoenzymes, which can enhance utilization of predigested substrates by mites^5,6^. *T. putrescentiae* feeds on fungi mycelia and fruit bodies and increases fungi transmission^7^. As so, it is a pest of mycology laboratories and edible fungi cultivation^8,9^. Storage mites are too small (adult body length, 280–420 *μ*m) to easily observe and their effects are often mistaken for diseases^10^.

Mites can disperse by wind (anemochory) or hitchhike on animals (phoresy). Mites in the phoretic stage must possess sensory capabilities needed for locating and identifying potential insect hosts. Once a potential host is found, they must have the mechanical ability to climb on and attach to the host insect^11^. However, the sensory capabilities of mites are virtually unknown^12^. Animals use semiochemicals or pheromones to communicate, and then have a series of behavioral responses, such as feeding, mating, avoidance, marking territory, finding prey, and host recognition^13,14^. In this process, several important chemosensory gene families are involved, including odorant binding proteins (OBPs), chemosensory proteins (CSPs), pheromone binding protein (PBPs), odorant degrading enzymes (ODEs) and chemical receptors, such as odorant (ORs), ionotropic (IRs) and gustatory receptors (GRs)^15^. Research on the molecular mechanism of chemoreception has demonstrated that volatile chemicals activate a single class of sensory neurons, which target the glomerulus and connect to projection neurons that respond exclusively to the chemicals^16^.

In this study, the GRs family was identified for the first time in *T. putrescentiae* by transcriptome sequencing. The GR genes were used for rapid detection of the storage mite from sample materials including bacteria, host fungi, competitive fungi, and fungus gnats using loop-mediated isothermal amplification (LMAP). LAMP of DNA is an alternative to PCR-based methods in food safety testing^17^ and a variety of other applications including detection of plant diseases and viruses in animals and plants^18–20^. Its advantages over PCR-based techniques include shorter reaction time, no need for specific equipment, high sensitivity and specificity, and comparably low susceptibility to inhibitors present in sample materials. This enables detection of the pathogens in sample materials without lengthy sample preparation.

## Results

### Identification of GR genes from *T. putrescentiae* transcriptome

A total of 84,756 unigenes with a mean length of 684 nt were assembled from about 48.91 million clean reads and 51.53 million raw reads (Table 1) by Illumina HiSeq2000. A total of 9,408 unigenes (11.10%) were larger than 1,000 nt in length. Of these genes, 38,169 unigenes (45.03%) were annotated in the NCBI NR database (e-value < 1e-5).

**Table 1:** The primers used in the LAMP assay.

Primer name	Sequence (5′-3′)	Num.
F3-Gr09	TGGGCAGCAAGAACAACG	18
B3-Gr09	TGGAGGAAGACGGTCCAG	18
FIP-Gr09	GCTGTTCGACTCGTCCACCAGCCTCTCGACGCTCTACTACG	42
BIP-Gr09	GTGTCACATTCCCTGCAACTGCGGTCATCAAAGTCGGCGAAG	43

**Figure 1:**
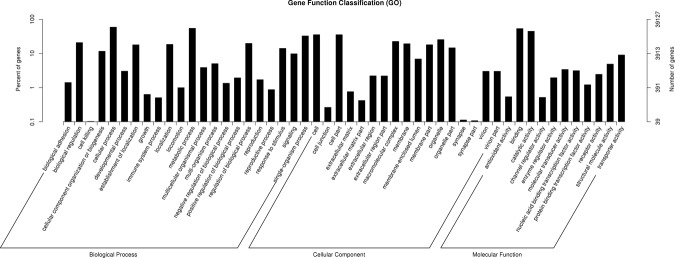
Gene Ontology classifications of the *T. putrescentiae* unigenes.

Gene ontology (GO) annotation of the unigenes was obtained using Blast2GO pipeline and a blastx search against NR. A total of 39,127 unigenes were assigned GO terms. In the biological process terms, the genes were mostly enriched in cellular and metabolic processes. In molecular function terms, molecular binding activity and catalytic activity were the most represented. In cellular component terms, cell, cell part, organelle, organelle part, macromolecular, membrane and membrane part were the most abundant (Fig. 1).

The unigenes related to gustatory receptors (GRs) were identified by keyword search of annotations in the seven databases. We identified distinct unigenes encoding one putative GR, and it was named TputGR1 following nomenclature previously established for *T. putrescentiae*^21^.

### Sequence analysis and phylogenetic tree construction

**Figure 2:**
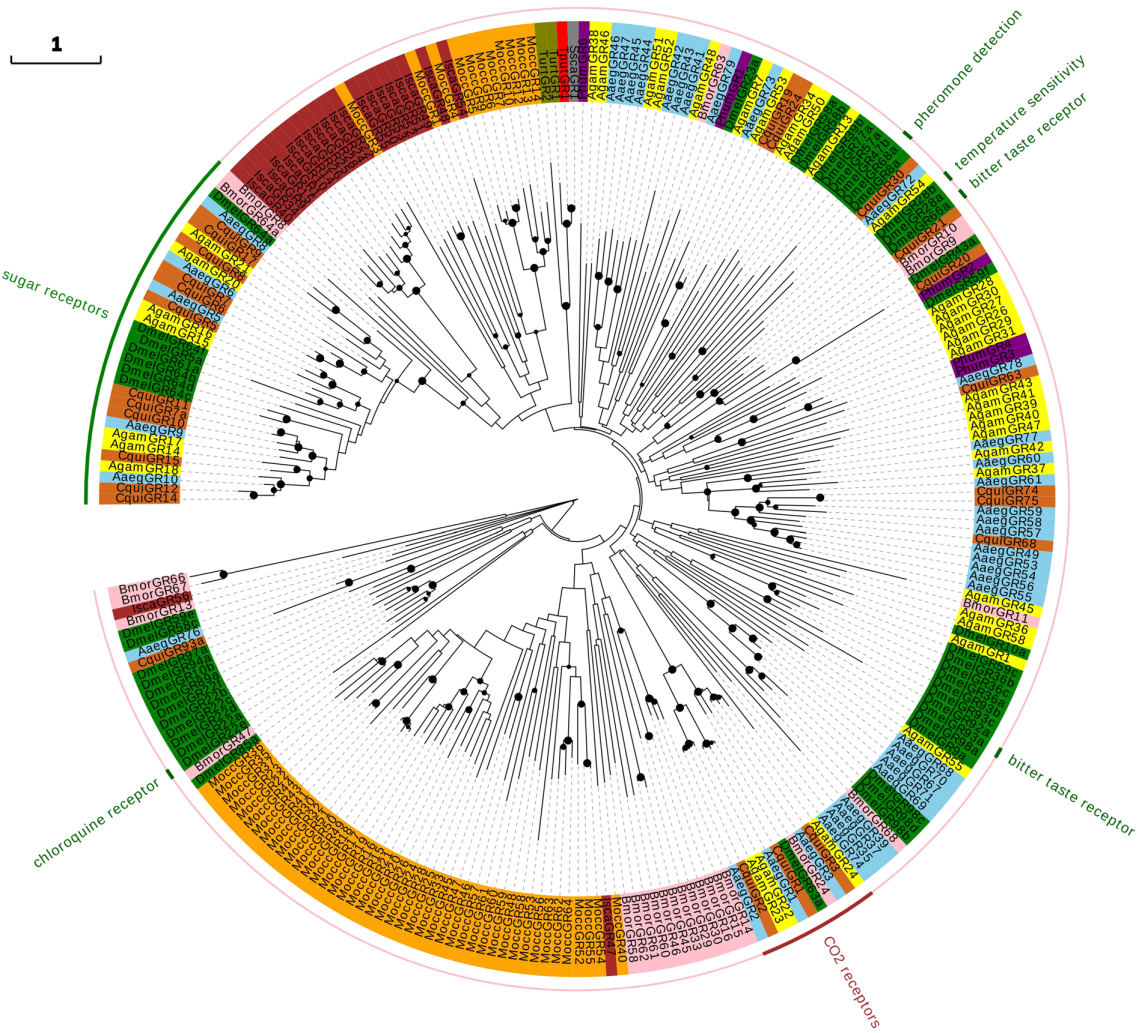
Unrooted phylogenetic tree of GR protein sequences from *T. putrescentiae* (red branches), *Drosophila melanogaster* (green branches), *Anopheles gambiae* (yellow branches), *Bombyx mori* (pink branches), *Aedes aegypti* (skyblue branches), *Culex quinquefasciatus* (chocolate branches), *I. scapularis* (brown branches), *M. occidentalis* (orange branches), *S. scabiei* (gray branches), *T. urticae* (olive branches) and *Pediculus humanus* (purple branches). Outer ring indicates the different groups and protein functions.

Sequence analysis confirmed the new one GR, TputGR1, as a 1330-bp long full-length transcript. TputGR1 had high homology with SccaGR1 of *Sarcoptes scabiei* and TurtGR1-2 of *Tetranychus urticae*, but low homology with other insect species (Fig. 2). Almost all GRs in Acari were orthologous with insects in the phylogenetic tree. Some GRs with related multi-functions were clustered into the same subgroup including 12 CO_2_ receptors in insects (including BmorGR11 and 24, AgamGR22, 23 and 24, DmelGR63a, AaegGR1-3 and CquiGR1-3)^22,23^, and 33 sugar receptors in insects (DmelGR5a, 61a, 64a-d and 64 f, BmorGR8 and 64a, AgamGR14-18 and 20-21, AaegGR5-6 and 8-10 and CquiGR7a, 5-15 and 17)^22,24,25^. No orthologs were found for 42 of the 54 GRs of *Metaseiulus occidentalis* and these were clustered into one subgroup. The remaining 11 of the 54 GRs of *M. occidentalis* were clustered together with all 21 GRs of *Ixodes scapularis* (Fig. 2). The expansion and diversification of the tick and mite GRs probably reflected the diverse evolutionary histories of chemosensory receptors in the Acari.

### Development of the loop-mediated isothermal amplification detection system

**Figure 3:**
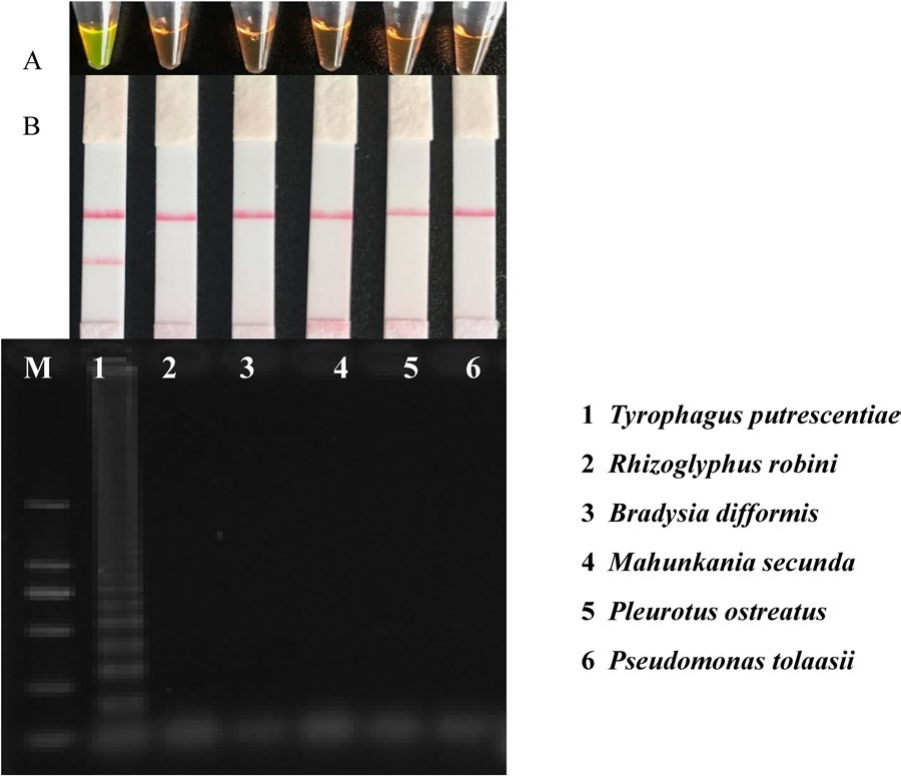
Correlation of color change and gel electrophoresis of the amplification LAMP results. The positive result produced yellowish green color in (**A**), two bands in (**B**) and a ladder-like pattern in (**C**). Samples 1–6 are *Tyrophagus putrescentiae*, *Rhizoglyphus robini*, *Bradysia difformis*, *Mahunkania secunda*, *Pleurotus ostreatus* and *Pseudomonas tolaasii*, respectively.

We developed of the loop-mediated isothermal amplification involved primer combinations with a high sensitivity and no false positives. A set of primers suitable for LAMP detection of *T. putrescentiae* (TP) was designated by F3-Gr09, B3-Gr09, FIP-Gr09, and BIP -Gr09 (Table 0). FIP-Gr09 and BIP -Gr09 were labeled with FAM and biotin, respectively. Primer combination, F3-Gr09, B3-Gr09, FIP-Gr09, and BIP -Gr09 gave a high sensitivity with no false positives and gel electrophoresis results below the strip graph confirmed the LAMP reaction corresponding to the results on the strip (Fig. 3B). The amplification of TputGR1 by LAMP produced a ladder-like pattern, whereas the PCR product was a specific DNA band. Agarose gel electrophoresis analysis indicated that the LAMP reaction did not detect the mushroom mites, *Rhizoglyphus robini* and *Mahunkania secunda*, the fungus gnat, *Bradysia difformis*, the mushroom pathogen, *Pseudomonas tolaasii*, and the mycelia of *Pleurotus ostreatus* host (all samples called TPDV). In addition to gel electrophoresis, both visual inspection and strip graph were performed to detect positive reactions and validate the LAMP results. Upon addition of the SYBR green I dye to tubes after the LAMP reaction, the color changed to yellowish green in the positive reactions (Fig. 3A). Similar results were found in the lateral flow strip detection (Fig. 3B).

### Optimization of loop-mediated isothermal amplification (LAMP)

**Figure 4:**
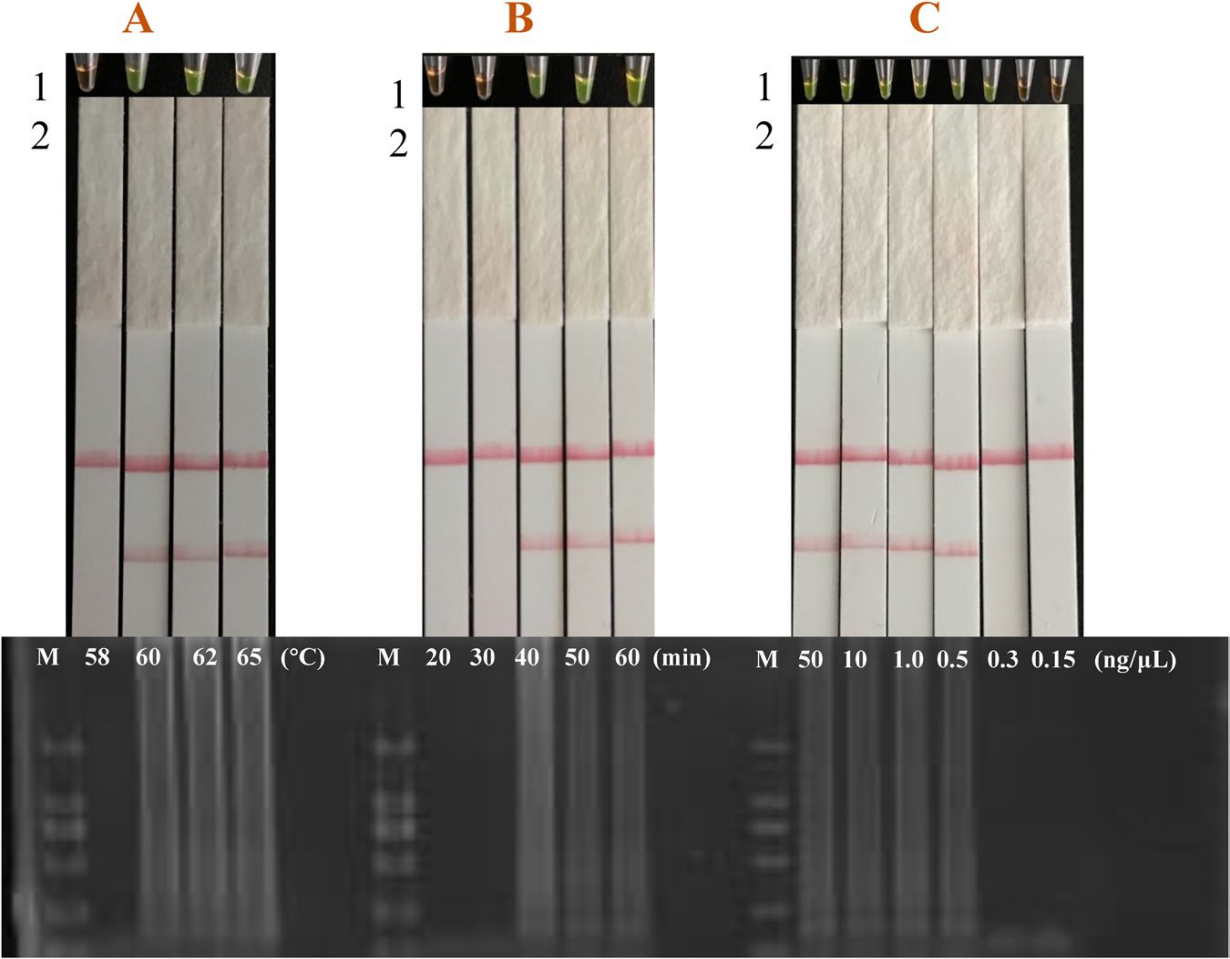
Optimization of LAMP. (**A**) LAMP with 58, 60, 62 or 65 °C of reaction temperature. (**B**) LAMP with 20, 30, 40, 50 or 60 min of reaction time. (**C**) LAMP with 50, 10, 1.0, 0.5, 0.3 or 0.15 ng/*μ*L of TP target. The results of the color change were also verified by gel electrophoresis (down) and lateral flow strip detection (up, 2).

LAMP assay optimizations focused on reaction temperature, reaction time and DNA concentration. A reaction temperature of 60 °C was selected for the LAMP assays (Fig. 4A), similar to a previous report^26^. The reaction time was extended by 10 and 20 min based on optimum experimental conditions to determine the relationship between reaction time and sensitivity. When LAMP reactions were incubated for 60 min, favorable results were obtained without nonspecific amplification (Fig. 4B). The sensitivity of LAMP was higher than that under 60 min (Fig. 4B). In addition, amplification results from the optimized LAMP assay correlated 100% with results on gel electrophoresis.

The total volume for the LAMP amplification was 25 *μ*L. The optimal concentration of each reagent was as follows: F3-Gr09, 0.1 *μ*M; B3-Gr09, 0.1 *μ*M; FIP-Gr09, 0.4 *μ*M; BIP-Gr09, 0.4 *μ*M; MgSO_4_, 0.5 mM; dNTP, 0.24 mM; and 1 × Bst DNA polymerase buffer and Bst DNA polymerase (8 U/*μ*L), 2 *μ*L. The assay could detect TP containing DNA target (50 ng/*μ*L) at 10^−1^ dilution after 60 min amplification.

### Evaluation and validation of LAMP-TP with clinical specimens

A total of 37 samples from swine suspected of having TP infections were collected from Chengdu (*Agaricus bisporus* host), Harbin (*Flammulina velutipes* host), and Jinan (*P. ostreatus* host). The samples were tested for validation of the LAMP-TP in TP detection. A no template control and three uninfected hosts, *P. ostreatus* (PO), *A. bisporus* (AB), and *F. velutipes* (FV), were used to evaluate and validate the LAMP-TP. Three hosts were detected positive by LAMP-TP (Figure S1). By contrast, all infected samples (No. 1 to 37) were detected positive by PCR using B3-Gr09 and F3-Gr09 primers (Figure S1). However, two samples (one from Chengdu and one from Jinan) were detected as positive by regular-PCR but negative by LAMP-TP.

## Discussion

Although a large number of the GR genes have been identified in insects, little is known about the olfactory system and mechanisms of Arachnida, which comprise over 100,000 named species, including spiders, scorpions, harvestmen, ticks, mites, and solifuges^27^. Our approach successfully identified low-expressing olfactory genes in a non-model mite species lacking genome sequences. We found that the GRs of three mites from the Acariformes were characterized into one subfamily, and low homology with the GRs from the Insecta. GRs are recognized as insect-specific chemosensory receptor families, expressed in gustatory receptor neurons (GRNs). Most GRs are classic seven transmembrane GPCRs involved in sweet and bitter taste reception and in the detection of non-volatile pheromones. However, there are four members of the GRs family that are expressed in ORNs, including two highly related GRs (GR21a and GR63a) that function in the detection of CO_2_^22,28^. We did not identify these members of the GRs family in mites, but provided information for comparative and functional genomic analyses of 11 species from the Insecta and Arachnida.

We also developed a rapid detection assay for *T. putrescentiae* based on the GR gene and the Lamp method. There have been few reports on the application of the LAMP method for mite detection. We developed a color method for detecting *T. putrescentiae* in samples of fungi using test strips or SYBR Green I dispensed with gel electrophoresis. The sensitivity of LAMP-TP was 0.5 ng/*μ*L and the assay duration was needed less than 60 min.

In summary, we established a LAMP-TP method specific for the detection of *T. putrescentiae*. This simple method has shorter detection time, high sensitivity, and strong specificity.

## Methods

### Mites

The *T. putrescentiae* strain studied originated from a population collected in 2012 on wood ear mushroom *Auricularia polytricha* in Feng County (Jiangsu, China). The mites were mass reared on *P. ostreatus* mycelia in the laboratory at 25 °C and 85% RH. The mites were cultured by methods reported previously^9^.

### Transcriptome sequencing

Total RNAs were extracted using SV Total RNA Isolation System (Promega, Madison, WI, USA) from adults (about 300 males and females mixed) according to manufacturer protocol. The cDNA library construction and the Illumina HiSeq2000 sequencing were conducted at Novogene Company (Beijing, China). The abundances of the unigenes in the transcriptomes were calculated by the RPKM (Reads Per Kilobase per Million mapped reads) method, using the formula: RPKM (A) = (1,000,000 × C × 1,000)/(N × L), where RPKM (A) is the expression abundance of gene A; C is the number of reads that are uniquely mapped to gene A; N is the total number of reads that are uniquely mapped to all genes and L is the number of bases on gene A. The RPKM method is able to eliminate the influence of different gene lengths and sequencing discrepancies in the calculation of transcript abundance^29^.

### Identification of chemoreceptor genes superfamily from the *T. putrescentiae* transcriptome

Transcriptome (without a reference genome) assembly was accomplished based on the left.fq and right.fq using Trinity with *min*_*kmer*_*cov* set to 2 by default and all other parameters set to default. Gene function was annotated based on the following databases: Nr (NCBI non-redundant protein sequences), Nt (NCBI non-redundant nucleotide sequences), Pfam (Protein family), KOG/COG (Clusters of Orthologous Groups of proteins), Swiss-Prot (A manually annotated and reviewed protein sequence database), KO (KEGG Ortholog database) and GO (Gene Ontology). Within these databases, GRs were identified by manually searching with the keywords “gustatory receptors”. The translated protein sequences of target genes were edited and subjected to BLAST online severs as well as further identification and analysis. TMHMM 2.0 (http://www.cbs.dtu.dk/services/TMHMM/) was used to predict transmembrane domains of candidate GRs.

### Sequence analysis and phylogenetic tree construction

GRs from *T. putrescentiae* identified in this study were aligned with the respective gene families from other arthropod species, *D. melanogaster*, *A. gambiae*, *B. mori*, *A. aegypti*, *C. quinquefasciatus*, *I. scapularis*, *P. humanus*, *S. scabiei*, *T. urticae* and *M. occidentalis* (Table S1). Genome sequence data and gene annotations were downloaded from public data repositories: *D. melanogaster* and *B. mori* from National Center for Biotechnology Information (ftp://ftp.ncbi.nih.gov/genomes), *A. gambiae*, *A. aegypti*, *C. quinquefasciatus*, *P. humanus* and *I. scapularis* from VectorBase (https://www.vectorbase.org/), M. occidentalis from Hoy *et al*. (2016)^12^. ORFs were identified and translated into amino acid sequences using Expasy software (http://web.expasy.org/translate/). The putative N-terminal signal peptides of all predicted olfactory genes were predicted by SignalP 4.0 server version (http://www.cbs.dtu.dk/services/SignalP/) with more than 70 amino acids before conducting the analyses^30^. All sequence alignments were performed using ClustalX2.1^31^. Phylogenetic trees were constructed by the neighbor-joining method with the Jones-Taylor-Thornton (JTT) amino acid substitution model as implemented in MEGA7.0 software and then edited in the Evolview server (http://evolview.codeplex.com/)^32^. Bootstrap support of tree branches was assessed by resampling amino acid positions 1000 times.

### Optimization of the LAMP detection system

#### DNA genome extraction

Six different total DNA samples were extracted from *T. putrescentiae* (TP), two other mushroom mites, *R. robini* and *M. secunda*, the fungus gnat, *B. difformis*, the mushroom pathogen, *P. tolaasii*, and from mycelia of the *P. ostreatus* host, using the Takara MiniBEST Viral DNA Extraction Kit Ver. 5.0 (Takara Biotechnology Co., Ltd., Dalian, China) according to the manufacturer instructions. The extracted DNAs were used as the templates in the LAMP assays.

#### LAMP procedure

We conducted a probe hybridization reaction experiment and determined the selected primers that exhibited no reaction during probe hybridization. Subsequently, the LAMP tests were conducted with different primer combinations based on the GR gene. The primers used in the LAMP assay to detect *T. putrescentiae* were designed in this study. The primers F3-Gr09, B3-Gr09, FIP-Gr09, and BIP-Gr09 (Table 1) were designed using online LAMP primer software PrimerExplorer (http://primerexplorer.jp/e/). The probes were labeled with FAM or biotin.

#### DNA genome extraction

LAMP reactions were performed in a total volume of 2 *μ*L and contained 1.0 *μ*L (10 pM) F3-Gr09 and B3-Gr09 primers, 4.0 *μ*L (10 pM) the FIP and BIP primers, 9.5 *μ*L (2.5 mM) of dNTPs, 2.5 *μ*L of 10X Bst DNA polymerase buffer, 0.5 *μ*L (8 U) of Bst DNA polymerase (New England BioLabs, Herts, UK), 2 *μ*L of template DNA, 0.5 *μ*L (100 mM) of MgSO _4_. The amplifications were performed at 65 °C for 60 min and were terminated by incubating the reactions at 80 °C for 10 min. In addition to gel electrophoresis, both visual inspection and strip graph were used to detect positive reactions and validate the LAMP results. Upon addition of the SYBR green I dye to tubes after the LAMP reaction, the color changed to yellowish green in the positive reactions. The products of LAMP were dropped onto the sample pad of the test strips (Ustar Biotech, Hangzhou, China). The test strips were observed after 1 min. Positive LAMP-TP was denoted by two red lines, with the top line for quality control and the bottom line for the test. Only the control line is negative. Otherwise, only the test line is invalid.

#### Sensitivity assay

The sensitivity of the LAMP-TP assay was evaluated using template concentrations from 0.15 ng/*μ*L to 50 ng/*μ*L. The genome dilution series were used in the LAMP assay and in PCR to determine the minimum detection concentrations for each assay.

#### Optimization of LAMP-TP

To detect *T. putrescentiae* from clinical specimens, a LAMP assay system was optimized. LAMP assay optimization included concentrations, reaction temperature and reaction time. Reaction temperatures selected for the LAMP assays ranged from 58 °C to 65 °C. The reaction time was extended by 10 to 30 min, based on the optimum experimental conditions to determine the relationship between reaction time and sensitivity. The amplification results from the optimized LAMP assay were detected by gel electrophoresis, color changes, and strip graph.

#### LAMP-TP used for detection in clinical specimens

We collected 37 samples from four edible mushrooms, *P. ostreatus*, *A. bisporus*, A. polytricha, and *F. velutipes*, apparently infected with the storage mite from 3 regions (Beijing, Harbin, and Jinan) of China to evaluate the LAMP-TP method. Presence of mites in the samples was determined by microscopic examination. Total DNAs were extracted from the 37 samples according to manufacturer protocol.

We applied the PCR LAMP to detect *T. putrescentiae* in the samples to verify the clinical potential of the method. The same samples were tested using a PCR method with the detection primers F3-Gr09/B3-Gr09.

## Electronic supplementary material

Supplementary Information
