# Supplementary material for: A Gustatory Receptor Used for Rapid Detection of Tyrophagus putrescentiae in Fungi Hosts
Source: Sci Rep. 2018 Jul 30;8:11425. doi: 10.1038/s41598-018-29729-4 (PMC6065420; doi:10.1038/s41598-018-29729-4)
Supplement: Supplementary file 1 — Supplementary Information [file 41598_2018_29729_MOESM1_ESM.pdf]

Supplementary Information

A Gustatory Receptor Used for Rapid Detection of *Tyrophagus putrescentiae* in Fungi Hosts

Shao-Xuan Qu[1, *, †], Xiao-Fei Wang[2, †], Hui-Ping Li[1], Xin Luo[1], and Lin Ma[1]

[1] Institute of Vegetable Crops, Jiangsu Key Laboratory for Horticultural Crop Genetic Improvement, Jiangsu Academy of Agricultural Sciences, Nanjing, 210014, China
[2] Cold Spring Harbor Laboratory, 1 Bungtown Rd, Cold Spring Harbor, New York, 11724, USA

*qusx@jaas.ac.cn
† These authors contributed equally to this work.
*corresponding: qusx@jaas.ac.cn

SUPPLEMENTARY FIGURES

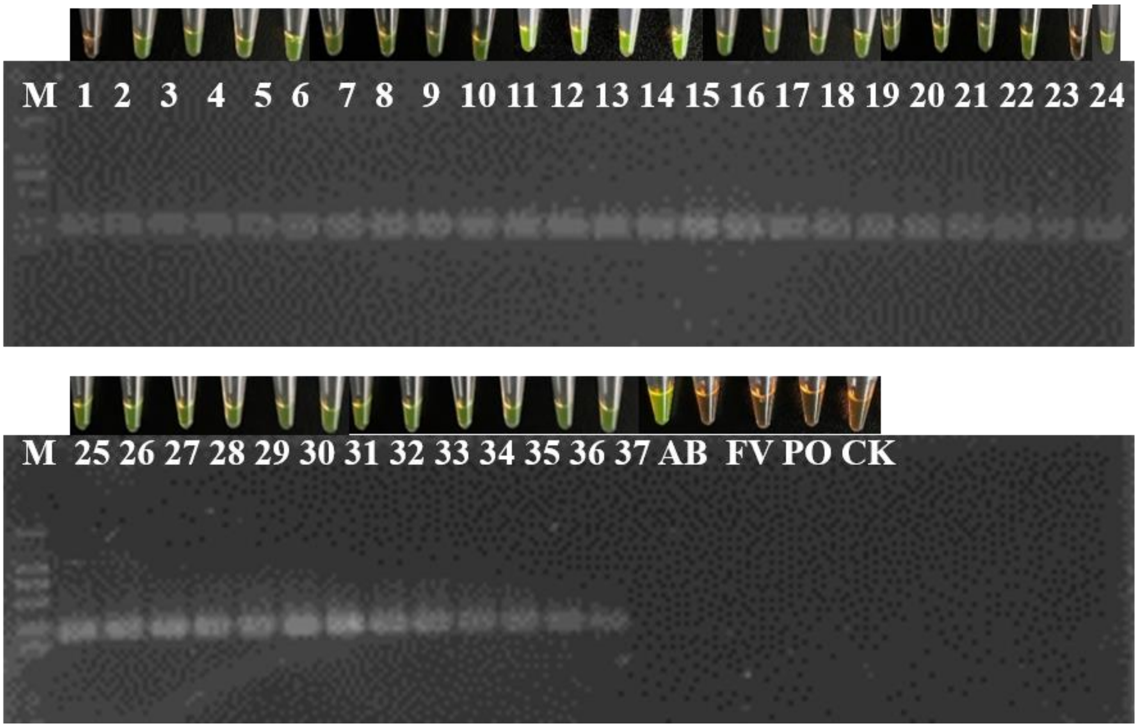

Fig. S1. Specificity of TP detection by LAMP-TP. 37 samples collected in Chengdu, Harbin, and Jinan were evaluated using the LAMP-TP assay. M: DL2000 DNA marker; No. 1 to 5 samples were from a *A. bisporus* host collected in Chengdu; No. 6 to 15 were samples from a *F. velutipes* host collected in Harbin; 16 to 37 samples were from a *P. ostreatus* host collected in Jinan; No template control (CK); Three uninfected hosts were *P. ostreatus* (PO), *A. bisporus* (AB), and *F. velutipes* (FV).

SUPPLEMENTARY TABLES

Table S1 Summary of chemoreceptor genes used in this study

Gene NO	NCBI/UniProtKB/Swiss-Prot Accession/ VectorBase gene label
DmelGR5a	Q9W497.2
DmelGR28b	Q9VM08.2
DmelGR28a	Q9VM09.2
DmelGR68a	Q9VTN0.2
DmelGR66a	Q9VSH2.3
DmelGR93a	Q9VD76.2
DmelGR63a	Q9VZL7.1
DmelGR61a	Q9W0M2.2
DmelGR64f	P83297.2
DmelGR22e	P58953.1
DmelGR64a	P83293.1
DmelGR8a	Q9W367.1
DmelGR39b	P58960.1
DmelGR22c	P58952.3
DmelGR92a	Q8IN58.2
DmelGR22d	P84181.1
DmelGR93b	Q8IN23.2
DmelGR22b	P84180.1
DmelGR58c	Q9W2B2.3
DmelGR36b	Q9VJF2.2
DmelGR93c	Q9VD74.2
DmelGR57a	Q9V969.1
DmelGR77a	Q8IPU5.1
DmelGR36c	Q8INZ2.1
DmelGR85a	Q8INM9.1
DmelGR22a	P58951.2
DmelGR59c	Q9W1U5.2
DmelGR58a	P58962.1
DmelGR36a	P58955.1
DmelGR22f	P58954.1
DmelGR58b	Q9W2B1.2
DmelGR59e	Q9W1N6.2
DmelGR59f	Q9W1N5.2
DmelGR94a	Q8IMZ5.1
DmelGR89a	Q9VEU0.2
DmelGR98a	Q9VB30.2
DmelGR98b	Q9VB26.2
DmelGR9a	Q8IRL8.1
DmelGR97a	Q8IMQ6.1

DmelGR98c	Q8IMN6.1
DmelGR98d	Q8IMN5.1
DmelGR47b	P58961.2
DmelGR10b	Q9VYZ2.1
DmelGR43a	Q9V4K2.3
DmelGR23a	P83292.1
DmelGR64b	P83294.1
DmelGR10a	P58950.1
DmelGR64c	P83295.1
DmelGR64d	Q9VZJ6.2
AgamGR24	AGAP001915
AgamGR22	AGAP009999
AgamGR45	AGAP007757
AgamGR28	AGAP006713
AgamGR38	AGAP001114
AgamGR37	AGAP001117
AgamGR7	AGAP009855
AgamGR46	AGAP007756
AgamGR47	AGAP005514
AgamGR43	AGAP005047
AgamGR42	AGAP001115
AgamGR41	AGAP001122
AgamGR34	AGAP006450
AgamGR14	AGAP006399
AgamGR39	AGAP001119
AgamGR40	AGAP001120
AgamGR36	AGAP001123
AgamGR13	AGAP002635
AgamGR53	AGAP002633
AgamGR55	AGAP006917
AgamGR58	AGAP001125
AgamGR18	AGAP003256
AgamGR16	AGAP003254
AgamGR15	AGAP003253
AgamGR27	AGAP006716
AgamGR17	AGAP003255
AgamGR26	AGAP006717
AgamGR29	AGAP006874
AgamGR30	AGAP006875
AgamGR31	AGAP006876
AgamGR23	AGAP003098
AgamGR20	AGAP003259
AgamGR54	AGAP004313

AgamGR51	AGAP001172
AgamGR52	AGAP001173
AgamGR48	AGAP001170
AgamGR50	AGAP001171
AgamGR1	AGAP004114
AgamGR21	AGAP003260
BmorGR9	NP_001124345.1
BmorGR8	NP_001124344.1
BmorGR68	NP_001233217.1
BmorGR67	NP_001233216.1
BmorGR60	NP_001124347.1
BmorGR45	NP_001124346.1
BmorGR47	ACD85126.1
BmorGR46	ACD85125.1
BmorGR66	BAK52798.1
BmorGR10	BAS18817.1
BmorGR16	DAA06379.1
BmorGR14	DAA06377.1
BmorGR13	DAA06376.1
BmorGR11	DAA06375.1
BmorGR63	DAA06395.1
BmorGR62	DAA06394.1
BmorGR61	DAA06393.1
BmorGR58	DAA06392.1
BmorGR33	DAA06386.1
BmorGR30	DAA06385.1
BmorGR29	DAA06384.1
BmorGR15	DAA06378.1
BmorGR24	XP_004931409.1
BmorGR64a	XP_004923090.1
CquiGR15	CPIJ016873
CquiGR14	CPIJ016219
CquiGR7a	CPIJ016218
CquiGR12	CPIJ016217
CquiGR11	CPIJ016216
CquiGR5	CPIJ0163214
CquiGR8	CPIJ015721
CquiGR63	CPIJ017565
CquiGR20	CPIJ000740
CquiGR6	CPIJ013956
CquiGR93a	CPIJ012214
CquiGR3	CPIJ007321
CquiGR1	CPIJ0006622

CquiGR21	CPIJ018209
CquiGR10	CPIJ016215
CquiGR9	CPIJ016211
CquiGR74	CPIJ014990
CquiGR68	CPIJ014987
CquiGR17	CPIJ013959
CquiGR7	CPIJ013957
CquiGR30	CPIJ014450
CquiGR75	CPIJ020301
CquiGR24	CPIJ019344
CquiGR2	CPIJ007380
CquiGR19	CPIJ017888
IscaGR1	ISCW006130
IscaGR50	ISCW014146
IscaGR54	ISCW023798
IscaGR25	ISCW014323
IscaGR24	ISCW014322
IscaGR53	ISCW022395
IscaGR52	ISCW022394
IscaGR34	ISCW010566
IscaGR35	ISCW020488
IscaGR10	ISCW011825
IscaGR9	ISCW011824
IscaGR40	ISCW012700
IscaGR28	ISCW011393
IscaGR22	ISCW011391
IscaGR26	ISCW011390
IscaGR56	ISCW007374
IscaGR55	ISCW002158
IscaGR15	ISCW003214
IscaGR14	ISCW003213
IscaGR49	ISCW002109
IscaGR48	ISCW002108
IscaGR51	ISCW000388
IscaGR47	ISCW0011591
PhumGR4	PHUM551510
PhumGR3	PHUM551500
PhumGR6	PHUM224030
PhumGR1	PHUM202890
PhumGR2	PHUM037560
AaegGR1	AAEL002380
AaegGR2	AAEL002167
AaegGR3	AAEL010058

AaegGR5	AAEL000043
AaegGR6	AAEL000012
AaegGR10	AAEL000082
AaegGR8	AAEL000069
AaegGR9	AAEL000075
AaegGR35	AAEL017415
AaegGR70	AAEL017432
AaegGR71	AAEL017307
AaegGR74	AAEL017476
AaegGR59	AAEL013200
AaegGR58	AAEL017324
AaegGR78	AAEL0017312
AaegGR72	AAEL017216
AaegGR73	AAEL010962
AaegGR57	AAEL010272
AaegGR56	AAEL010278
AaegGR55	AAEL010274
AaegGR54	AAEL017364
AaegGR49	AAEL017488
AaegGR67	AAEL017158
AaegGR68	AAEL017527
AaegGR69	AAEL017441
AaegGR79	AAEL017486
AaegGR60	AAEL017450
AaegGR61	AAEL017202
AaegGR77	AAEL007940
AaegGR39	AAEL017206
AaegGR37	AAEL017235
AaegGR43	AAEL017182
AaegGR42	AAEL017449
AaegGR41	AAEL006500
AaegGR46	AAEL017092
AaegGR45	AAEL006494
AaegGR44	AAEL017569
AaegGR47	AAEL017408
AaegGR76	AAEL009545
AaegGR53	AAEL010279
MoccGR1	-
MoccGR3	-
MoccGR4	-
MoccGR5	-
MoccGR6	-
MoccGR7	-

MoccGR9	-
MoccGR10	-
MoccGR12	-
MoccGR13	-
MoccGR14	XP_003746805.1
MoccGR15	-
MoccGR16	-
MoccGR17	-
MoccGR18	-
MoccGR19	-
MoccGR20	XP_003743441.1
MoccGR21	XP_003743441.1
MoccGR22	-
MoccGR23	-
MoccGR24	-
MoccGR25	-
MoccGR26	-
MoccGR28	-
MoccGR30	-
MoccGR32	-
MoccGR35	-
MoccGR36	-
MoccGR37	-
MoccGR40	-
MoccGR41	-
MoccGR43	-
MoccGR44	-
MoccGR45	-
MoccGR46	-
MoccGR47	-
MoccGR48	-
MoccGR49	-
MoccGR50	-
MoccGR51	-
MoccGR52	-
MoccGR53	-
MoccGR54	-
MoccGR55	-
MoccGR56	-
MoccGR57	-
MoccGR58	-
MoccGR59	-
MoccGR60	-

MoccGR61	-
MoccGR62	-
MoccGR63	-
MoccGR64	-

Notes: Protein sequences of *Metaseiulus occidentalis* are provided in Hoy et al 2016.

Table S2 List of putative GRs and IRs identified in *T. putrescentiae*

Name	Size (aa)	Sequence (ORF)
TputGR1	443	MDQRKGFQAQMPNRDAFTIPKLAYEFYFKAFGLISTGLD FGQRYGPIKQDKTATNLKRFYCQLVCLMLWFFAIRGFVL MFIGDREIQLMLGDLTGFWNDYRMYYLMPTFYYSLQTA IIATIFLRNEQELAWLVPFVSVKQMQTNSIRTAKYDTNNH ERRTQVTIIINNLIVLVATGMVGSLYTLTAYENMDQYTFR LFVPWIGVQCIWIFFMAGINMFTMTYFNLVCLILSNRFK QVCKDIEALAESDPGPLGSKNNALSTLYYEHNEICELVD ESNSFWQSFIFFNYMCHIPCNCYALYNLFFADFDDLLAIV TWTVFLHTILFLAFISLSAADVSAEAHSPYTALHTLSLLQ LPIDLEVNMSTFLHRVRGPTIGFSCLDLFVITNASISNTIA AVASYFLIVADFSRSTAAANAAEKREEAARAASALNITTT AATPTAGS
